# Anti-tumor effects of the American cockroach, *Periplaneta americana*

**DOI:** 10.1186/s13020-017-0149-6

**Published:** 2017-09-12

**Authors:** Yanan Zhao, Ailin Yang, Pengfei Tu, Zhongdong Hu

**Affiliations:** Modern Research Center for Traditional Chinese Medicine, School of Chinese Materia Medica, Beijing University of Chinese Medicine, No.11 North Third Ring Road, Chaoyang District, Beijing, 100029 China

**Keywords:** *Periplaneta americana*, Anti-tumor, Apoptosis, Angiogenesis, Immunity

## Abstract

Since the incidence of cancer has been on the rise due to increasing exposure to various carcinogenic factors in recent years, cancer has gradually become the first killer to the health of human beings. A growing attention has been paid to anti-cancer effects of traditional Chinese medicine (TCM) with low toxicity and good efficacy. As a kind of TCM, *Periplaneta americana* (*P. americana*) has a good effect on clinical application, and its anti-tumor effects has been increasingly well studied. In this review, the research progress on the anti-tumor effects of *P. americana* was summarized. The main mechanisms of its anti-tumor effects include suppression of tumor cell growth, induction of cell cycle arrest and tumor cell apoptosis, inhibition of angiogenesis, enhancement of immunity, and reversal of tumor drug resistance. This review aims to provide an overview of the research on anti-tumor effects of *P. americana* and aids in its further application as an anti-tumor drug.

## Background

As one of the leading causes of death in the world, cancer has been the focus of extensive research [1]. According to GLOBOCAN 2012, about 14.1 million new cancer cases and 8.2 million deaths occurred in 2012 worldwide [2]. Meanwhile, there were approximately 3.4 million cancer patients in China in 2012, and the number of cancer deaths was about 2.46 million [3]. Cancer can seriously threaten the patients’ quality of life and their survival. Surgery, radiotherapy, and chemotherapy are widely used for the treatment of cancer in the world. However, these methods cannot effectively change the causal interaction of individual factors related to the pathological process. Therefore, it is difficult to completely inhibit tumor recurrence and metastasis. Long-term treatment with these methods may facilitate drug resistance and cause serious side effects in patients [4]. Traditional Chinese medicine (TCM) has a well-established history of high efficiency and low toxicity [5–8]. Tumor treatment with TCM is carried out through the overall regulation of the body [9]. In recent years, cancer therapy using TCM with characteristics of multi-level, multi-link, and multi-target has garnered increasing attention [10–13].

*Periplaneta americana*, more commonly known as the American cockroach, is the part of Insecta class, Dictyoptera order, and Blattidae family. It is one of the largest, strongest, oldest, and most successful breeding insect groups [14]. The dried worms or fresh adults of *P. americana* are often used as a TCM drug [15]. Its taste is salty and acrid, and its nature is cold. These features can promote blood circulation, remove blood stasis, help digestion, aid in detoxification, and induce diuresis for treating edema. *P. americana* can also be used to treat infantile malnutrition, tonsillitis, body phlegm, carbuncles, sore throat, and insect bites. Modern pharmacological research has revealed that *P. americana* has anti-tumor effects, and is able to enhance immunity, promote tissue repair, stabilize blood pressure, improve microcirculation, protect the liver, and act as an anti-inflammatory, anti-bacterial, and anti-viral agent as well as an analgesic and antioxidant [15, 16].

### Clinical application of *P. americana*

Active ingredients isolated from *P. americana* have been developed into clinical drugs in China [17], such as “Xiaozheng Yigan Tablet”, “Kangfuxin Liquid”, “Ganlong Capsule”, and “Xinmailong Injection”. “Xiaozheng Yigan Tablet” is an oral tablet with potent anti-tumor effects and anti-bacterial activity. It has been reported to reduce liver inflammation, promote the recovery of liver function, and reduce the degree of liver fibrosis in patients with hepatitis B virus (HBV) infection [18]. Moreover, it has been revealed in a study with 66 cases of primary liver cancer treated with “Xiaozheng Yigan Tablet” that the level of alpha-fetoprotein was reduced and survival time of patients was prolonged [19]. “Kangfuxin Liquid” has been used in clinic for more than 20 years. The main functions of “Kangfuxin Liquid” include eliminating inflammation, reducing swelling, promoting cell proliferation and growth of new granulation tissue, and promoting organism recover. It is mainly applied for stomach and duodenal ulcer, pressure sores, wounds, and burns. Though the curative effect of “Kangfuxin Liquid” is good, its obvious side effects have not been found [20]. “Ganlong Capsule” has a good anti-hepatitis B virus effect and is characteristic of low price, convenient administration, and little side effect [21]. “Xinmailong Injection” has a wide range of therapeutic effects on the cardiovascular system, including improving microcirculation, expanding pulmonary vessels, diuresis, anti-arrhythmic, inhibiting free radical damage, and anti-atherosclerosis. Clinical trials have demonstrated that “Xinmailong Injection” has good therapeutic effect in congestive heart failure and chronic pulmonary heart disease, and the total effective rate is more than 80%. In addition, no obvious untoward reaction was found during treatment [22]. The clinical application of these drugs continue to increase due to limited adverse reactions [16]. Especially, the effect of *P. americana* on anti-tumor and immune regulation has attracted widespread attention and increasingly become the research focus [23].

### Chemical constituents of *P. americana*

Numerous studies have shown that the main chemical constituents of *P. americana* include pheromones, proteins, fatty acids and esters, amino acids, alkaloids, alkanes, polysaccharides, isoflavones, cockroach oil, and peptides [16, 24–31]. It was reported that the 50 components of *P. americana* were separated and identified, most of which were unsaturated fatty acids and esters [26]. Ten cyclic peptides were isolated and purified from *P. americana*, eight of which were isolated for the first time [27]. Another study identified 23 compounds in *P. americana* including 16-hydro-7-hexadecenoic acid lactone (35.98%), fatty acids and esters (26.62%), aliphatic aldehyde, stigmast-4-ene-3-one, alkanes, palmitic acid, and linoleic acid [28]. 19 compounds were also separated and identified from *P. americana*, which mainly included alkanes, octadecadienoic acid, and octadecadienoic alcohol [29]. *P. americana* contains more than 16 amino acids, including 7 human essential amino acids and two human semi-essential amino acids [30]. The 70% ethanol extract of *P. americana* contains amino acid, alkaloid, fatty acids and esters, and pheromones [25]. More than 50 neuropeptides have been identified from *P. americana,* including allatostatins, pyrokinins, fraps, kinins, and periviscerokinins [31]. In addition, *P. americana* also contains polysaccharides, cockroach acid, cockroach oil, allergens, chitosan, cytochromes A, B, and C [16, 27].

### Pharmacological activity of *P. americana*

A large number of studies have shown that *P. americana* has anti-tumor, anti-bacterial, anti-viral, anti-radiation, detumescence, analgesic, and anti-inflammatory effects. In addition, *P. americana* was shown to protect the liver, promote blood vessel growth, aid in tissue repair, improve microcirculation, and enhance immunity. *P. americana* also possesses a high antioxidant capacity demonstrated by the clearance of 2,2-diphenylpicrylhydrazyl and OH free radicals [15, 32]. In recent years, the anti-tumor activity of *P. americana* has become a research focus.

### Anti-tumor effects of *P. americana*

Accumulating evidences have revealed the anti-tumor effects of *P. americana* on a variety of cancer cells. Herein, we summarized the reported the mechanisms underlying the anti-tumor effects of *P. americana*.

#### Inhibition of tumor cell growth

Studies have shown that some TCM drugs can inhibit the growth of tumor cells in vitro and in vivo [12, 33, 34]. These drugs can be used at various stages of tumorigenesis. Mechanistically, these treatments can inhibit the synthesis of DNA, RNA, and proteins, and block the energy metabolism of tumor cells [35]. A previous study has showed that CII-3 from the *P. americana* caused cytotoxicity in two human lung cancer cell lines [36]. Moreover, *P. americana* extract inhibited the growth of three human reproductive system cancer cell lines and three human respiratory system tumor cell lines [37, 38]. In addition, *P. americana* extract suppressed the growth of three human and mouse leukemia cell lines [39]. The 60% ethanol fraction of *P. americana* organic extracts (PAE60) inhibited tumor growth in S180 tumor-bearing mice by 72.62%. Moreover, PAE60 was determined against 12 human cancer cell lines, and it could effectively inhibited the growth of HL-60, KB, CNE, and BGC823 cells with IC_50_ values <20 µg/mL [23].

#### Cell cycle arrest

Cell cycle is a complex process involving multiple factors, such as cyclins, cyclin-dependent protein kinases, and cell cycle-dependent protein kinase inhibitors [40, 41]. The abnormal expression of cyclins and cyclin-dependent protein kinases, and loss of cyclin-dependent protein kinase inhibitors can cause uncontrolled cell proliferation and tumor growth [42]. It has been shown that *P. americana* extracts can inhibit the growth of progesterone receptor-negative endometrial cancer cells by blocking the cell cycle via up-regulation of p53 expression and down-regulation of C-erbB-2 expression [43]. *P. americana* extract could arrest the cell cycle of human lung cancer cells H125 in the S phase [44]. Human gastric cancer BGC-823 cells exhibited the cell cycle arrest at G2/M phase in the presence of “Kangfuxin Liquid” that consists of the refined active constituents of *P. americana* [45]. Moreover, *P. americana* extract inhibited the growth of Lewis lung carcinoma (3LL) cells in mice and induced cell cycle arrest in G0/G1 phase [46].

#### Induction of apoptosis

Apoptosis is a process of programmed cell death, which plays a critical role in cancer development and therapies [47–49]. Multiple genes are involved in apoptosis in cancer cells, such as pro-apoptotic proteins Fas, Bax, p53 and anti-apoptotic proteins Bcl-2, c-myc [50, 51]. Many natural products can induce apoptosis in various human cancer cells, such as gambogic acid, ursolic acid, vinca alkaloids, and camptothecins [8, 52]. *P. americana* extract inhibited the proliferation of human hepatoma cells by inducing apoptosis and reducing the mitochondrial membrane potential, up-regulating Bax, Caspase-9, and Caspase-3 expression, and down-regulating Bcl-2 expression [53]. *P. americana* extracts induced apoptosis in Lewis lung carcinoma (3LL) cells through up-regulation of Fas, Fas receptor (FasR), and p53 gene expression and down-regulation of Bcl-2 expression [54]. In addition, a study revealed that *P. americana* extract induced apoptosis in human hepatocellular carcinoma SMMC-7721 cells via the mitochondrial pathway [55].

#### Anti-angiogenic effect

Nutrients and oxygen supplied by the vasculature are essential for tumor growth and metastasis. Thus, angiogenesis plays a vital role in tumorigenesis [56, 57]. Vascular endothelial growth factor (VEGF), a major contributor to angiogenesis, promotes the proliferation and migration of endothelial cells, and increases vascular permeability [58, 59]. *P. americana* polypeptides significantly inhibited tumor growth, decreased tumor microvessel density (MVD), and reduced VEGF expression [60]. *P. americana* extract significantly inhibited the tumor growth of H22 tumor-bearing mice and reduced VEGF levels in mice serum [61]. These evidences indicate that the anti-tumor effect of *P. americana* is probably related to angiogenesis inhibition.

#### Enhancement of immunity

As an important guarantee for the body health, immunity is closely related to the stability of the internal environment. The homeostasis of the organism is destroyed when the immunity of the organism declines, which contributes to occurrence and spread of tumors [62, 63]. Thus, improving body immunity can achieve anti-tumor effect [64]. Tumor necrosis factor alpha (TNF-α) is a multifunctional cytokine with a crucial role in apoptosis, cell survival, and immunity [65, 66]. TNF-α is mainly secreted by monocytes and macrophages and exerts its biological functions through binding to specific receptors on the cell surface and activating intracellular distinct signaling pathways [67]. *P. americana* polypeptide extracts had a strong inhibitory effect on S180 and H22 tumor-bearing mice. The extracts increased the spleen index and thymus gland index of tumor-bearing mice, promoted the proliferation of T lymphocytes, enhanced the phagocytotic function of macrophages, and up-regulated the levels of IL-2, IL-6, IL-12, and TNF-α [68]. Moreover, *P. americana* extracts markedly inhibited tumor growth without causing toxicity of immune organs in S180 tumor-bearing mice, which may be related to an increase in TNF-α in the serum of tumor-bearing mice [69]. *P. americana* could increase the CD4/CD8 ratio of peripheral blood in mice with low immunity [70]. Therefore, *P. americana* extract showed significant anti-tumor activity that might be related to an enhanced immune function in vivo.

#### Reversal of drug resistance

Chemotherapy is one of the most common and effective methods in cancer treatment. The advancement of molecular biology, biochemistry, and genetic engineering techniques have led to remarkable achievements in the research and development of anti-tumor drugs. However, drug resistance has become a major obstacle for treatment of cancer [71, 72]. Drug resistance is a complex process involving multiple factors in cancer therapy. Thus, it is urgent and important to improve drug resistance in cancer treatment. There is evidence that *P. americana* extract effectively reversed the drug resistance of human hepatoma cells by targeting the multidrug resistance protein (MRP), breast cancer resistance protein (BCRP), and P-glycoprotein (P-gp) [73]. Additionally, human hepatoma HepG2/ADM cell line has biological characteristics of multi-drug resistance, and *P. americana* extract could inhibit the growth of HepG2/ADM cells along with reversal of drug resistance [74].

## Conclusion

**Fig. 1:**
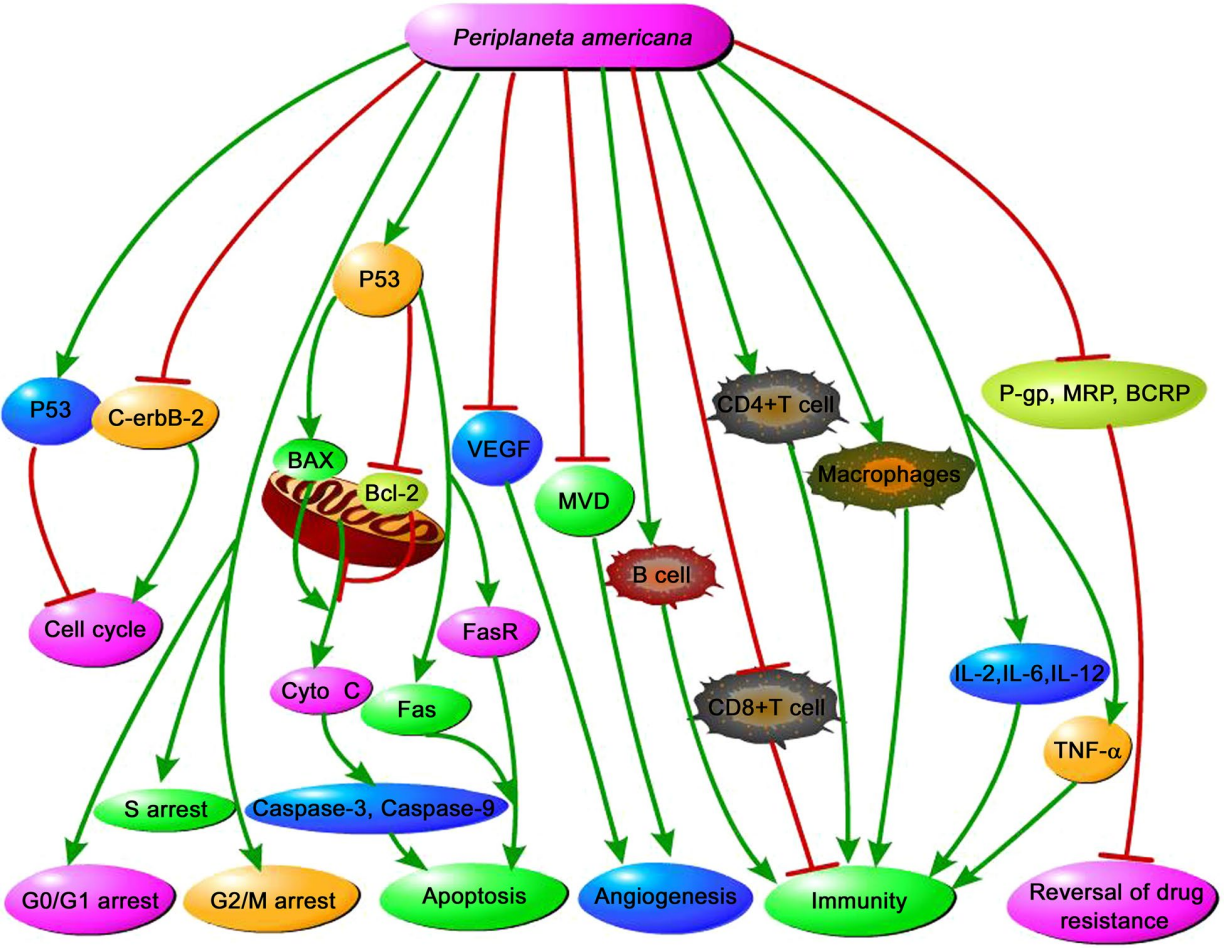
Diagraphic illustration of mechanisms of anti-tumor effects of *P. americana*. *P. americana* extract inhibited cell proliferation via p53- and C-erbB-2-mediated cell-cycle arrest in progesterone-receptor negative endometrial cancer cells. *P. americana* extracts induced cell-cycle arrest of Lewis lung carcinoma (3LL) cells and human lung cancer cells H125 at G0/G1 phase and S phase, respectively. “Kangfuxin Liquid” induced cell-cycle arrest of human gastric cancer cells BGC-823 at G2/M phase. *P. americana* extract decreased the ratio of Bcl-2 to BAX by increasing p53 and upregulated Fas and FasR expression, which induced apoptosis of cancer cells. *P. americana* may depress angiogenesis by inhibiting VEGF expression and microvessel density (MVD). *P. americana* extract activated lymphocytes, increased the CD4/CD8 ratio of peripheral blood, prompted T- and B-lymphocytes proliferation, and modulated cytokines release, including TNF-α, IL-2, IL-6, and IL-12. In addition, the extract effectively reversed the drug resistance of human hepatoma cells (HepG2/ADM) by targeting P-gp, MRP, and BCRP

*Periplaneta americana* extract has been widely applied in China as an alternative medicine against diseases. The above studies have shown that the anti-tumor effects of *P. americana* are attracting more and more attention. This review aimed to provide a clear picture regarding the anti-tumor effects and the underlying mechanisms of *P. americana*. The reported mechanisms of anti-tumor effects of *P. americana* mainly involve inhibition of tumor cell growth, induction of cell cycle arrest and apoptosis, suppression of angiogenesis, enhancement of immunity, and reversal of drug resistance (Fig. 1). However, the specific active constituents and precise mechanisms underlying the anti-cancer activities of *P. americana* remain uncertain. Thus, it is an important research topic to optimize the extraction process and search for the best technological conditions for further isolation and purification of the anti-tumor constituents of *P. americana*. Additionally, the mechanisms of anti-tumor effects of *P. americana* remain to be further identified. More exploration remain to be performed, such as effects of *P. americana* on cancer metastasis or autophagy. It may be promising to conduct further investigation of PAE60 on the identification of active chemical constituents and relevant pharmacological mechanisms. To be sure, further exploration of anti-cancer drug from *P. americana* will provide potent scientific basis for clinical use of *P. americana* and contribute to the development of novel anti-cancer drugs with high efficiency and low toxicity.

## Abbreviations

TCM: traditional Chinese medicine
VEGF: vascular endothelial growth factor
TNF-α: tumor necrosis factor-α
*P. americana*: *Periplaneta americana*
MVD: microvessel density
HBV: hepatitis B virus
PAE60: 60% ethanol fraction of *Periplaneta americana* organic extracts
FasR: Fas receptor
MRP: multidrug resistance protein
BCRP: breast cancer resistance protein
P-gp: P-glycoprotein
